# Diagnostic potential of IL6 and other blood-based inflammatory biomarkers in mild traumatic brain injury among children

**DOI:** 10.3389/fneur.2024.1432217

**Published:** 2024-07-11

**Authors:** Anne-Cécile Chiollaz, Virginie Pouillard, Céline Habre, Michelle Seiler, Fabrizio Romano, Fabian Spigariol, Céline Ritter Schenk, Christian Korff, Fabienne Maréchal, Verena Wyss, Lyssia Gruaz, Joan Montaner, Sergio Manzano, Jean-Charles Sanchez

**Affiliations:** ^1^Department of Internal Medicine, Faculty of Medicine, University of Geneva, Geneva, Switzerland; ^2^Pediatric Neurology Unit, Department of the Woman, Child and Adolescent, Geneva University Hospitals, Geneva, Switzerland; ^3^Division of Radiology, University Hospitals of Geneva, Geneva, Switzerland; ^4^Department of Pediatric Emergency, University Children's Hospital Zurich, Zürich, Switzerland; ^5^Division of Pediatric Emergency Medicine, Department of Pediatrics, Inselspital, Bern University Hospital, University of Bern, Bern, Switzerland; ^6^Department of Pediatric Emergency, Neuchâtel Hospital (RHNE), Neuchâtel, Switzerland; ^7^Department of Pediatrics, Fribourg Hospital HFR, Fribourg, Switzerland; ^8^Platform of Pediatric Clinical Research, Department of Woman, Child and Adolescent, Geneva University Hospitals, Geneva, Switzerland; ^9^Neurovascular Research Group, Institute of Biomedicine of Seville, IBiS/Virgen Macarena University Hospital/CSIC/University of Seville, Seville, Spain; ^10^Department of Pediatric Emergency, Geneva University Hospitals and Faculty of Medicine, University of Geneva, Geneva, Switzerland

**Keywords:** biomarkers, cytokines, diagnosis, emergency, mild traumatic brain injury (mTBI), pediatric

## Abstract

**Objectives:**

Inflammatory biomarkers, as indicators of biological states, provide a valuable approach for accurate and reproducible measurements, crucial for the effective management of mild traumatic brain injury (mTBI) in pediatric patients. This study aims to assess the diagnostic utility of blood-based inflammatory markers IL6, IL8, and IL10 in children with mTBI, including those who did not undergo computed tomography (CT) scans.

**Methods:**

A prospective multicentric cohort study involving 285 pediatric mTBI patients was conducted, stratified into CT-scanned and non-CT-scanned groups within 24 h post-trauma, alongside 74 control subjects. Biomarker levels were quantitatively analyzed using ELISA. Sensitivity and specificity metrics were calculated to determine the diagnostic efficacy of each biomarker.

**Results:**

A total of 223 mTBI patients (78%) did not undergo CT scan examination but were kept in observation for symptoms monitoring at the emergency department (ED) for more than 6 h (in-hospital-observation patients). Among CT-scanned patients (*n* = 62), 14 (23%) were positive (CT+). Elevated levels of IL6 and IL10 were found in mTBI children compared to controls. Within mTBI patients, IL6 was significantly increased in CT+ patients compared to both CT– and in-hospital-observation patients. No significant differences were observed for IL8 among the compared groups. IL6 yielded a specificity of 48% in identifying CT– and in-hospital-observation patients, with 100% sensitivity in excluding all CT+ cases. These performances were maintained whether IL6 was measured within 6 h or within 24 h after the trauma.

**Conclusion:**

The inflammatory marker IL6 emerges as a robust biomarker, showing promising stratification value for pediatric mTBI patients undergoing CT scans or staying in observation in a pediatric ED.

## Introduction

Traumatic brain injury (TBI) can strike us all and belong to major global health problems. In children, annual incidence of TBI ranges between 47 and 280 per 100,000 children (1). Mild TBI (mTBI), defined by a Glasgow Coma Scale (GCS) score of 13–15, account for the majority of TBI (80%) (2, 3). Increasing incidence in pediatric subpopulations is observed, with children and adolescents representing almost 70% of mTBI cases (4, 5).

mTBIs are heterogeneous, presenting a variety of symptoms such as confusion or disorientation, loss of consciousness (LOC), post-traumatic amnesia, headache, vomiting, and other transient neurological abnormalities. mTBIs might increase the risk of developing emotional and neurocognitive disorders in later life, which can affect daily activities (6). Indeed, TBIs lead to a complex pathological sequence characterized by the initial trauma, and subsequent injuries to the central nervous system (CNS). This cascade of neuroinflammatory events aimed at removing damaged tissue while preserving neurological function (7). While neuroinflammation is recognized as a key mediator of the secondary brain injury following TBI, the dynamics of the immune response to TBI, occur within minutes after the injury. This process progresses from the onset of acute inflammation, initiated by danger signals and early inflammatory mediators, including cytokines and chemokine (8, 9).

Pediatric brain responses after injury differ from adult, due to developmental reasons, like the maturity of the immune system (10). The stage of brain development at the time of the injury, triggering an inflammatory response leading to immune dysregulation, may result in a variable age-dependent response. Given the significant role of the neuroinflammatory response in determining the extent of injury following mTBI, we would expect variations between the immature and adult brain. Therefore, investigating inflammatory mediators after mTBI in children, is necessary to the understanding of these acute and chronic inflammatory responses, and to have the appropriate knowledge for their specific management. However, a paucity of literature exists with regards to the pediatric population. Most cytokines responses have been studied in adult and severe TBI and there is limited knowledge regarding biomarkers relevance in children (11–14).

Interleukin-6 (IL6) is a pleiotropic cytokine, involved in neuronal regeneration and playing a role in pro-inflammatory response during the acute phase post-trauma. IL6 is one the best characterized inflammatory markers in severe and moderate adult TBI (15). The chemokine interleukin-8 (IL8) has also been widely studied for its significant contribution to disease-associated processes, including tissue injury, fibrosis, and angiogenesis (16). Together with IL6, IL8 has been found to be increased in the acute stage of severe adult TBI (17). Another cytokine, interleukin-10 (IL10), known for its anti-inflammatory properties and mainly expressed in macrophages and B cells (18), has also been largely investigated in adult TBI (19–21). However, our understanding of the cytokines and chemokines released after mild forms of TBI remains limited.

The presence of intracranial injuries (ICI) in mTBI patients is typically diagnosed using cranial computerized tomography (CT) scans. Although most are negative, a small proportion (<10%) might present an ICI, such as hemorrhage, that may require surgical intervention (22). In adults, several proteins have been investigated as stratification markers in mTBI patients. Scandinavian and French clinical guidelines have approved the use of S100 calcium binding protein B (S100b), and glial fibrillary acidic protein (GFAP), both astrocyte proteins, to the purpose of identifying patients with low risk of ICI (23–26). While theses biomarkers are promising, one of the ongoing challenges is the difficulty in assessing their clinical practice as diagnostic biomarkers due to their non-optimal specificity, and difficult definition of cut-off levels for the moment (27, 28).

Most of the children suffering mTBI (65–90%) are not scanned, because they are particularly at risk of cancer secondary to ionizing radiation (29–32), and care should be taken to avoid unnecessary exposure. They are kept under observation in the emergency department (ED) to monitor their symptoms for 6–24 h (in-hospital-observation without CT patients). This observation time is however stressful for children and parents, and cost consuming for the health care system. Management of mTBI children in both the acute and chronic phases after their trauma is a relevant clinical challenge, that might be improved by the investigation of blood-based biomarkers. Previously, we demonstrated in a prospective multicenter pediatric cohort of TBI patients, that GFAP, HFABP and, S100b, when sampled within 6 h after the trauma could be promising in children suffering from mTBI (33). Although these biomarkers show potential, further studies are required to validate their clinical utility, along with further investigation to identify other promising diagnostic markers.

To better identify and discriminate children with or without ICI after mTBI, the measurement of cytokines and chemokines released after mTBI, might provide additional readily diagnostic tools for clinicians.

Our aim was to assess in the same pediatric mTBI cohort, the performances of IL6, IL8, and IL10, to predict the absence of ICI with high sensitivity and avoid both unnecessary CT scans and prolonged stays in the ED.

## Materials and methods

### Study population

This prospective multicenter study was conducted in Switzerland, between October 2020 and February 2023 (t-BIOMAP, CCER-2020-01533). Children with TBI were recruited in five swiss pediatric EDs which all received institutional review board approval. The study was registered in www.clinicaltrials.gov (ID: NCT06233851) and conducted in accordance with Good Clinical Practice guidelines, and provisions of the Declaration of Helsinki. All patients or their legal representative were given oral and written information about the study at the ED, and written consent was obtained.

Inclusion criteria were: newborns to 16-year-old children with TBI within ≤ 24 h with, in addition, either (1) GCS ≤ 14; or (2) A GCS equal to 15 and one of the following symptoms: loss of consciousness during < 30 min, post traumatic amnesia < 24 h, persistent headaches, irritability, three or more episodes of vomiting, vertigo or dizziness, confusion, post-trauma convulsion; or (3) Sign for basal skull fracture; or (4) High kinetic TBI (traffic accident or fall of >0.9 m if < 2 years old and of 1.5 m if ≥ 2 years old). Exclusion criteria were: patient already included in another clinical study involving pharmacological treatment, proof of alcohol or other substances intoxication, history of TBI within the last month prior to consultation, history of epileptic seizures within the last month, Down syndrome, encephalitis, or meningitis. In one hospital, a control group was also recruited. Inclusion criteria were: newborn to 16-year-old children without a recent (<1 month) TBI, with scheduled blood sample in the ambulatory care unit. Exclusion criteria were identical to those of the TBI group.

Blood was drawn as soon as possible, and no later than 24 hours after trauma. The study did not interfere with any medical decisions, such as whether to perform a CT scan or to place the patient under observation.

Study data were collected and managed using REDCap electronic data capture tools hosted at Hôpitaux Universitaires de Genève (HUG) (34, 35).

The following results presented here pertain only to a subset of the recruited children: those with a mild TBI, selected based on the following criteria: GCS 14 or 15. In this specific population, biomarkers can have a real impact on patient management.

### CT scan analysis

The same pediatric radiologist (CH) reviewed all CT scans without knowledge of the clinical evaluation, biomarkers results, or local CT conclusions. A CT scan was considered positive for intracranial injury (ICI) if it showed any of the following: intracranial hemorrhage or contusion, cerebral edema, traumatic infarction, diffuse axonal injury, shearing injury, sigmoid sinus thrombosis, midline shift of intracranial contents or signs of brain herniation, skull diastasis, pneumocephalus, or skull fracture depressed by at least the width of the skull table [according to the Pediatric Emergency Care Applied Research Network (PECARN) criteria (36)].

### Blood biomarker analysis

Serum samples were promptly collected upon arrival at the ED and subsequently centrifuged and stored at −80°C. Blood levels of cytokines IL6, IL8 and IL10, were measured all at once using ELISA V-plex Proinflammatory Panel 1 (Human) antibodies (Meso Scale Diagnostics, Rockville, MD, USA).

Lower limits of detection (LLoD) were, respectively, 0.06 pg/mL with a calibration range of 0.06–488 pg/mL for IL6, 0.07 pg/mL LLoD and 0.07–375 pg/mL calibration range for IL8, 0.04 pg/mL LLoD and 0.04–233 pg/mL calibration range for IL10. Lower limits of quantification (LLoQ) were 0.633 pg/mL, 0.591 pg/mL, and 0.298 pg/mL for IL6, IL8, and IL10, respectively. All kits were used in accordance with the manufacturers’ instructions. Duplicate control serum was measured on each plate and, intra-and inter-plate coefficients of variation were below 20%.

### Outcome measures

The primary outcome was the presence of ICI on CT scans. Diagnostic values of blood-based biomarkers were assessed to rule-out the need of unnecessary CT scans and shorten the length of stay in ED observation.

### Statistical analysis

Patients were categorized into three groups: those who remained in observation without undergoing a CT scan (in-hospital-observation), those who underwent a CT scan with a negative result (CT–), and those who underwent a CT scan with a positive result (CT+). Statistical analysis was conducted using R^1^ in RStudio.^2^ The concentrations of cytokines IL6, IL8, and IL10 were normalized using their median values as correction factors to eliminate potential systematic bias related to the pre-analytical phase in each of the five involved centers. The reference center was chosen based on the cytokine median closest to that of the control group. Biomarker concentrations were compared between groups as follows: (1) in-hospital-observation without CT and CT– versus CT+ groups, as the primary objective, and (2) CT– versus CT+ groups, as a secondary objective. Differences between groups were assessed using the non-parametric Mann–Whitney U test, given the Kolmogorov–Smirnov test indicating a non-normal distribution for all cytokines (*p*-value <0.05). Kruskall-Wallis test (non-parametric ANOVA) was employed for comparisons involving more than two groups. The chi-squared test was utilized for the statistical analysis of clinical data, with statistical significance established at a *p*-value <0.05. Potential risk factor of the presence of extracranial injuries (ECI) was analyzed in a multivariable logistic regression model, with unadjusted and adjusted odds ratios, confidence intervals and *p*-values.

Cytokine levels are illustrated in box-and dot-plots on a log10 Y-scale, aiding in visual interpretation. The ability of IL6, IL8, and IL10 levels to classify patients according to their CT result group was evaluated using receiver operating characteristic (ROC) curves, employing the pROC package in R. The analysis focused on identifying optimal performance metrics to ensure all patients with intracranial injury (ICI) were correctly classified, aiming for the highest specificity when sensitivity was set to 100%, thereby achieving a 100% negative predictive value (NPV).

## Results

### Clinical parameters

Serum samples of 359 children were analyzed. This included 285 mTBI patients and 74 controls without head trauma. A total of 223 mTBI patients (78%) did not undergo CT scan examination but were kept in observation for symptoms monitoring at the ED for more than 6 h (in-hospital-observation patients) (Table 1). Within CT-scanned patients (*n* = 62), 14 (23%) were positive (Tables 1, 2). Mean age was 8 years old in all groups (standard deviations (SD) of 4.93–4.41–4.81–4.58 for respective controls, mTBI in-hospital-observation, CT– and CT+ groups). A wide range of ages from newborn to teenagers was observed (from 1-month to 16 years old). A high majority of the patients (93% of in-hospital-observation, 73% of CT–, and 71% of CT+ patients) had a GCS of 15 with associated symptoms. Loss of consciousness was the most frequent symptoms in mTBI patients (Table 1).

**Table 1:** Clinical parameters and biomarkers expression in controls and mTBI patients (with or without CT scan) – within 24 h post trauma.

		mild TBI *n* = 285			
	Ctrl (*N* = 74)	In-hospital obs. *n* = 223 (78%)	CT *n* = 62 (22%)		
			CT − *n* = 48 (77%)	CT + *n* = 14 (23%) (5% of mTBI)	*p*-value
Age (yo)					
Mean (SD)	8.7 (4.93)	8.6 (4.41)	8.7 (4.81)	8.6 (4.58)	0.997
Median [Min, Max]	8.8 [0.1, 16.8]	9.1 [0.2, 15.9]	8.2 [0.1, 16.0]	8.2 [0.9, 15.0]	
Sex, n (%)					
Boys	40 (54%)	124 (56%)	31 (65%)	10 (71%)	0.431
Severity of injury, n (%)					
GCS 14	–	16 (7%)	13 (27%)	4 (29%)	<0.001
GCS 15	–	207 (93%)	35 (73%)	10 (71%)	
Symptoms at inclusion, n (%)					
Loss of consciousness	–	161 (72%)	27 (56%)	10 (71%)	0.25
Post-traumatic amnesia	–	69 (31%)	13 (27%)	1 (7%)	0.259
Persistent headaches	–	68 (31%)	10 (21%)	5 (36%)	0.447
More than 3 episodes of vomiting	–	39 (18%)	6 (13%)	3 (21%)	0.653
Vertigo	–	21 (9%)	2 (4%)	0 (0%)	0.405
Confusion	–	26 (12%)	9 (19%)	1 (7%)	0.121
Convulsion	–	3 (1%)	2 (4%)	0 (0%)	0.211
Extracranial injuries (ECI), n (%)					
Either body lesions or fractures	–	23 (10%)	13 (27%)	4 (29%)	0.003 1.000 (CT−/CT+) 0.229 (CT−& Obs/CT+)
Skull fracture (on CT), n (%)					
Simple skull fracture (not PECARN criterion)	–	−	10 (21%)	11 (79%)	<0.001
Time laps TBI-blood (hours)					
Mean (SD)	–	6.11 (4.29)	6.90 (5.74)	9.57 (7.69)	0.313
Median [Min, Max]	−	5.00 [1.00, 23.0]	5.00 [1.00, 24.0]	8.00 [2.00, 24.0]	
IL6 (pg/mL)					
Mean (SD)	1.07 (2.32)	1.79 (5.62)	5.04 (11.9)	5.89 (8.70)	<0.001
Median [Min, Max]	0.393 [0.109, 16.0]	0.782 [0.0771, 77.6]	1.79 [0.115, 62.7]	2.62 [0.856, 32.3]	
*Missing*	*1 (1.4%)*	*2 (0.9%)*	*0 (0%)*	*0 (0%)*	
IL8 (pg/mL)					
Mean (SD)	19.7 (10.3)	18.2 (47.3)	166 (827)	183 (636)	<0.001
Median [Min, Max]	16.8 [3.90, 59.8]	9.72 [1.84, 565]	15.6 [4.35, 5,640]	12.6 [3.96, 2,390]	
*Missing*	*1 (1.4%)*	*2 (0.9%)*	*0 (0%)*	*0 (0%)*	
IL10 (pg/mL)					
Mean (SD)	1.02 (3.59)	1.00 (2.18)	3.64 (13.6)	1.56 (2.37)	<0.001
Median [Min, Max]	0.401 [0.127, 30.9]	0.411 [0.077, 17.4]	0.932 [0.063, 92.2]	0.640 [0.205, 9.37]	
*Missing*	*1 (1.4%)*	*2 (0.9%)*	*0 (0%)*	*0 (0%)*	

Mild TBI, mild traumatic brain injury; Ctrl, control; in-hospital obs., in-hospital observation patients for more than 6 h, and without CT; CT, computed tomography; yo, years old; GCS, Glasgow coma scale; SD, standard deviation; Min, minimum; Max, maximum.

*p* values correspond to the Kruskall-Wallis test (non-parametric ANOVA) comparison among the fourth group of the table.

**Table 2:** PECARN criteria for positive CT.

	CT + (*n* = 14)
1. Intracranial hemorrhage or contusion	12 (86%)
Sub-arachnoidal hemorrhage	1 (7%)
Epidural hemorrhage	2 (14%)
Intra-parenchymal hemorrhage	2 (14%)
Sub-dural hemorrhage	10 (71%)
2. Cerebral oedema	0 (0%)
3. Traumatic infraction	0 (0%)
4. Diffuse axonal injury or shearing injury	0 (0%)
5. Sigmoïd sinus thrombosis	0 (0%)
6. Midline shift of intracranial contents or signs of brain herniation	1 (7%)
7. Diastasis of the skull	3 (21%)
8. Pneumocephalus	7 (50%)
9. Skull fracture depressed by at least the width of the table of the skull	3 (21%)

Most of the included children experienced isolated mTBI. However, there were also children with extracranial injuries (ECI) reported. Among patients who underwent CT scan examinations, 13 (27%) with CT– and four (28%) with CT+ also had major body lesions or fractures, accounting for ECI. This proportion was significantly different in in-hospital-observation patients without CT, with only 23 (10%) patients presenting ECI. Fractures were the most common reason for ECI among patients who underwent CT scans (Table 1).

Except for the GCS score and the presence of ECI due to fractures, no significative differences in clinical parameters were observed when comparing mTBI patients with or without a CT scan (Table 1). However, among patients who underwent a CT scan examination, all clinical parameters described in Table 1 showed non-significant differences.

### Age correlation

Age correlation was investigated in the control group without head trauma. Spearman correlation analysis revealed that IL6 showed a slight but significant positive correlation with age (*r* = 0.24, with *p* value 0.04), IL10, on the other hand, exhibited a significant negative correlation with age (*r* = −0.54, with *p* value <0.0001), while IL8 did not show a significant correlation with age (*r* = −0.13, with *p* value 0.27) (Supplementary Data, Figure 1).

### Intracranial injuries on CT

The proportions of patients presenting the PECARN criteria, which are those used to define a CT+ result, are detailed in Table 2. Mostly, intracranial hemorrhages (86%) and pneumocephalus (50%) were observed among CT+ scans. Sub-dural hemorrhage was the most frequent type of hemorrhage, present in 71% of patients with hemorrhages. Additionally, out of the 14 patients with a CT+ result, 11 (79%) also presented simple skull fractures visible on the CT scan, which do not meet the criteria outlined by PECARN.

### Biomarkers blood levels and performances

The time between head trauma and blood sampling was slightly delayed in CT+ group (mean of 9 h versus 6 h, in the two other groups in-hospital-observation and CT–); however, this difference was not significant (*p* value =0.31).

**Figure 1:**
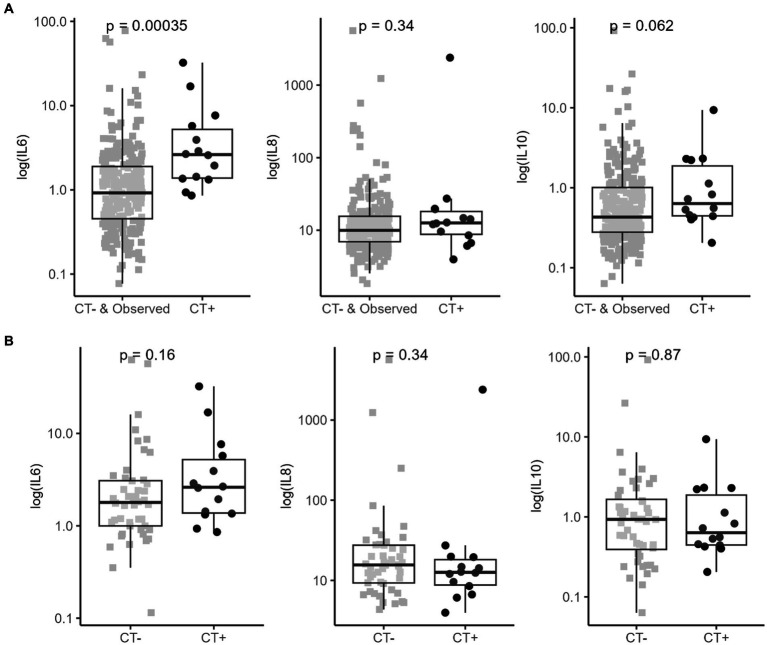
Cytokines serum concentration in mTBI patients (within 24 h). **(A)** Biomarkers expression within CT- or in-hospital-observation patients and CT+ mTBI patients (CT-scanned and observed without CT [>6 h at ED] patients). **(B)** Biomarkers expression within CT- and CT+ mTBI patients (only CT-scanned patients). Positive CT is based on PECARN criteria. Box plots represent median and IQR for compared groups; dot plots represent for each patient log scaled biomarker’s concentration. The analysis was carried out using a Mann–Whitney U test (shown *p*-value).

Means with SDs, and medians with minimum and maximum of cytokines levels in each group of patients, were reported in Table 1. Significant differences in biomarkers expression were observed when comparing all groups (*p* value <0.001) (Table 1). Blood concentration of IL6 and IL10 were increased in mTBI children compared to controls. Within mTBI patients, IL6 was significantly increased in CT+ patients compared to both CT– and in-hospital-observation patients. However, IL10 and IL8 levels were not significantly different among children with mTBI (Figures 1A,B and Supplementary Data, Tables 1, 2).

**Figure 2:**
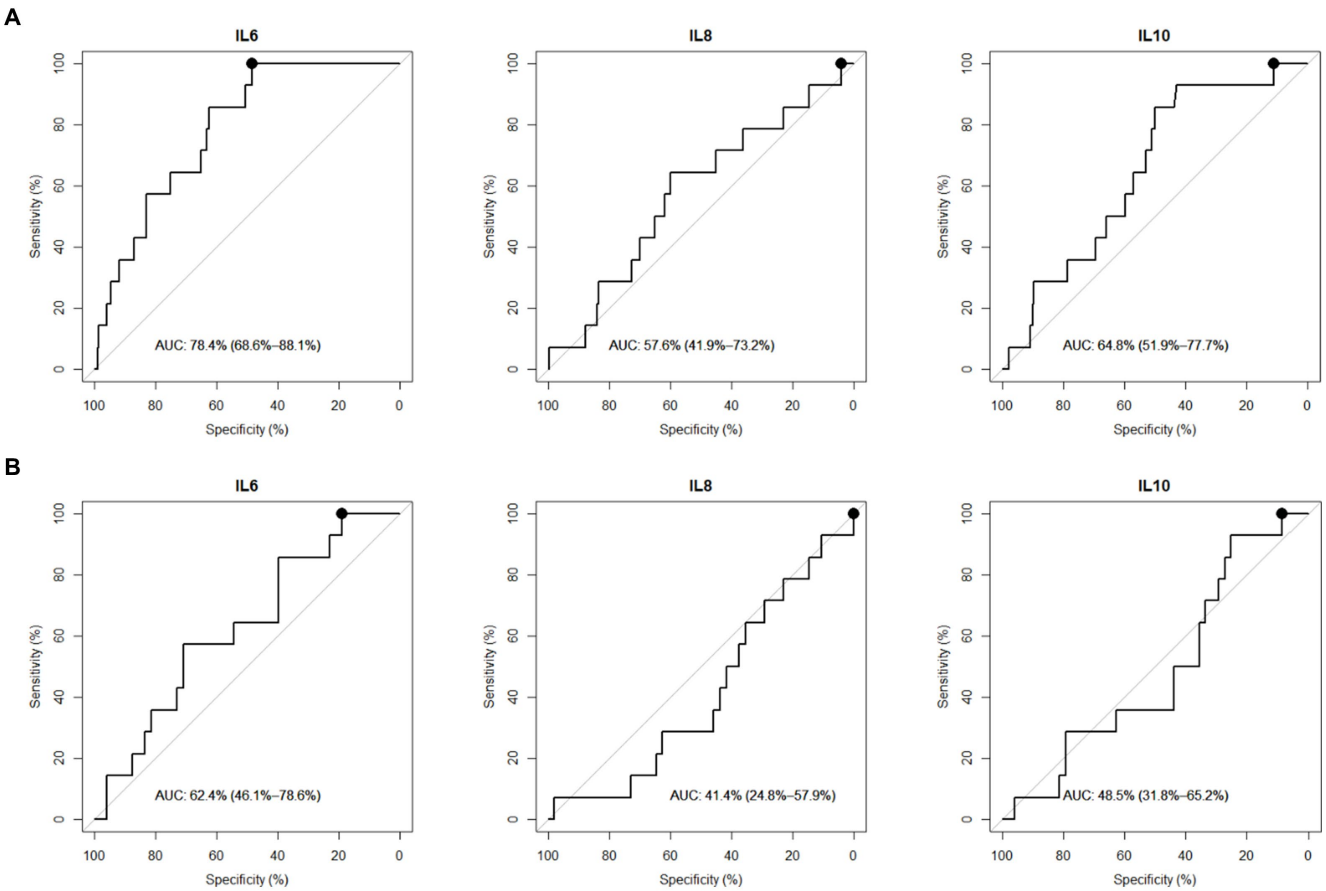
Cytokines diagnostic performances to classify mTBI patients (within 24 h). **(A)** Diagnostic performances within CT- or in-hospital-observation patients and CT+ mTBI patients (CT-scanned and observed without CT [>6 h at ED] patients). **(B)** Diagnostic performances within CT- and CT+ mTBI patients (only CT-scanned patients). Receiver Operating Characteristic (ROC) Curves in mTBI patients. AUC = Area Under the Curve with 95% confidence interval. Performances were investigated at 100% sensitivity and corresponding highest specificity (black round on ROC curve).

ROC curve analysis for the diagnostic performance of these three cytokines allowed to select the best specificity when sensitivity was set at 100%, aiming to exclude all children with ICI (Figures 2A,B). Under these conditions, IL6 yielded a specificity of 48% [95% IC: 43–54%], correctly identifying CT– and in-hospital-observation patients with 100% sensitivity while excluding all CT+ cases (Table 3A). This performance was maintained when focusing only on patients sampled within 6 h after trauma (212 mTBI patients out of the total 285 analyzed), with IL6 still achieving 50% specificity [95% IC: 43–57%] for 100% sensitivity (Supplementary Data, Table 3A). In contrast, IL10 and IL8 showed 11% specificity [95% IC: 7–15%] and 4% specificity [95% IC: 2–6%] respectively, in identifying CT– and in-hospital-observation patients when sensitivity was set at 100% (either at 24 or 6 h after the trauma).

**Table 3:** Cytokines best performances to rule-out mTBI patients (within 24 h).

(A)				
Variable	Sensibility (%) (95%CI)	Specificity (%) (95%CI)	Threshold (pg/mL)	AUC (95%CI)
IL6	100 (100–100)	48.33 (42.53–54.13)	0.85	78.36 (68.6–88.1)
IL10	100 (100–100)	11.11 (7.46–14.76)	0.20	64.79 (51.9–77.7)
IL8	100 (100–100)	4.09 (1.79–6.39)	3.91	57.59 (41.9–73.2)

(B)				
Variable	Sensibility (%) (95%CI)	Specificity (%) (95%CI)	Threshold (pg/mL)	AUC (95%CI)
IL6	100 (100–100)	18.75 (9.03–28.47)	0.83	62.35 (46.1–78.6)
IL10	100 (100–100)	8.33 (1.45–15.21)	0.20	48.51 (31.8–65.2)
IL8	100 (100–100)	0.00 (0.00–0.00)	-Inf	41.37 (24.8–57.9)

(A) Best performances to rule out a maximum CT− and in-hospital-observation patients, while all CT+ patients have been identified (CT-scanned and observed without CT [>6 h at ED] patients). IL6 yields the best performances (100% SE–48% SP).

(B) Best performances to rule out a maximum of CT− patients, while all CT+ patients have been identified (only CT-scanned patients). IL6 yields the best performances (100% SE–19% SP).

AUC, area under the curve.

IL6 performances were further investigated in only isolated mTBI patients (244 isolated mTBI out of the total 285 patients). IL6 concentration was still significantly increased in the CT+ group (*p* value <0.001), and its performance was even greater (100% sensitivity and 68% specificity [95% IC: 62–74%]) (Supplementary Data, Tables 4, 5).

The multivariable logistic regression model evaluating the presence of ECI as a risk factor on IL6 performances in correctly classifying patients, showed an adjusted odds ratio of 3.32 [95% IC: 0.79–14.02%], and was not significant (p value = 0.079) (Supplementary Data, Table 6).

For the second studied comparison, when focusing solely on identifying CT– versus CT+ patients, IL6 demonstrated 19% specificity [95% IC: 9–28%] at 100% sensitivity. Under these conditions, IL10 and IL8 specificities were lower than 10% at 100% sensitivity. These performances were similar either at 24 or 6 h after the trauma (Table 3B and Supplementary Data, Table 3B).

## Discussion

Our study underscores the potential of the cytokine IL6 as new candidate for triaging the need for CT scans in pediatric mTBI. In this multicenter pediatric cohort, IL6 achieved 100% NPV for CT+ cases, and identified one in two patients with CT– or without CT but with good evolution after in-hospital-observation, when sampled within twenty-fours after the trauma. As previously published on this cohort (33) GFAP, HFABP and S100b, respectively, reached 52, 41 and 39% specificity at 100% sensitivity, to also rule-out the need of unnecessary CT scans and shorten the length of stay in observation for patients without CT scan, when sampled within 6 h after the trauma. Their AUC values derived from ROC curve analysis, were not significantly different when compared to IL6 in the same conditions (Delong test, *p* value = 0.08). All these biomarkers therefore shown equivalent discriminatory capacity, even though they capture different pathophysiological mechanisms. Their use in combination represents the preferred option to further improve the discriminatory performance, knowing that TBI might lead to a variety of brain lesions leading to different proteins releases.

In this work, we underline that compared to previous brain blood-biomarkers, IL6 kept interesting performances while sampled within longer time-window after the trauma (up to 24 h). In our cohort, this concerned a quarter of the children who had their blood drawn later than 6 h after their trauma.

The measurement of blood-based inflammatory markers in mTBI is a recent topic of interest. IL6 is a well know biomarker in diseases of the central nervous system (CNS), such as Alzheimer’s Disease (AD), Parkinson’s Disease (PD) or Huntington’s disease (HD), due to its consistent upregulation whenever neuroinflammation is expected (37). Therefore, standard analytical technics for the measurement of IL6 in blood samples are already available in routine hospital laboratory medicine, which is an important step, toward its use in clinical diagnosis for mTBI patients.

In addition to IL6, which is primarily pro-inflammatory, we also investigated IL10, an anti-inflammatory cytokine, for its potential role in pediatric mTBI. Previous research had demonstrated IL10’s capacity to predict the absence of ICI on CT in adults with mTBI (19). Its observed elevation post-injury might reflect the body’s effort to counterbalance the acute inflammatory response. However, regarding our results, its performances to predict the absence of ICI on CT were not transposable from adult to children suffering mTBI. IL10 might be critical in modulating the inflammatory response after injury, potentially reducing the risk of secondary brain injury (38). This suggests that IL10 could better represent a prognostic indicator for recovery trajectory.

Finally, we investigated IL8’s involvement in pediatric mTBI, to explore the chemotactic response that recruits neutrophils to the site of injury, indicating a different aspect of the inflammatory process compared to IL6 and IL10. Its role in the acute phase of inflammation, particularly in the context of blood–brain barrier permeability and the subsequent infiltration of immune cells into the brain, could be crucial for understanding the early pathophysiological changes following mTBI. In our cohort, the observed elevated levels of IL8 in CT-scanned patients might signal a heightened inflammatory response, possibly correlating with more severe clinical presentations. However, IL8 did not provide any value in discriminating among CT+ and other children (CT– and in-hospital-observation children) with mTBI.

The performances of these three cytokines were also deciphered to distinguished between CT+ and CT– patients only. In this condition, the cytokines were less specific to identify CT– children while detecting all CT+ children. This could be attributed to a potential bias resulting from the inclusion of only those children who underwent scanning, identified by clinicians as higher risk according to clinical decision rules. We previously highlighted that clinical investigations into biomarkers for pediatric mTBI should aim not only to distinguish CT– patients, as it is usually done in adult mTBI, but also to discharge those kept under observation for hours in the ED (in-hospital-observation patients) to discard clinically important intracranial injuries. This methodology is closer to real life emergency management of mTBI children.

Furthermore, mild TBIs are not so mild, and neuroinflammation sequences due to the initial hit of the brain might be presents, while no ICI are identified on CT. For this reason, it is important to better consider and include the non-scanned children, kept in-observation at ED after their trauma, as they can also experience neuroinflammation without necessarily leading to ICI. Theses pathological events can lead to post-concussion symptoms (PCS), impairing the good recovery of the children (39). Considering the long-term outcome after mTBI, is another deep challenge in the patient’s management, where biomarkers should also definitely take a place. Despite it all, the present study was focusing on the acute phase after mTBI, and aimed to bring relevant biomarkers, such as IL6, toward decision’s tools for pediatricians in ED.

A well-recognized limitation of inflammatory blood-based markers is that they are non-specific to brain injury. Inflammation is present in response to almost any disease involving cellular damage. In TBI, ECI are sources of systemic inflammation markers (40), and are therefore known to be confounding factors, that might impact the specificity of the evaluated biomarkers. In our study, IL6 was found to be increased in all mTBI patients with ECI, which was an expected result for this pro-inflammatory cytokine, potentially released in the blood from other systemic inflammation. To distinguish neuroinflammation from systemic sources, considering orthopedic injured patients in addition to the healthy control patients might be relevant. In our cohort, in only isolated mTBI patients, IL6 and IL10 were still significantly increased in CT+ patients compared to both in-hospital-observation and CT– patients. Blood-based biomarkers, and especially inflammatory biomarkers can be influenced by medication use. Children’s current medications were monitored in the patient study record, and blood sampling was performed at the time of admission, prior potential medication intake at the ED.

Although promising, clinical utility of individual markers is not yet sufficient, and combinations of markers into panel, with both novel and already better-established markers, might improve their predictive performances (41, 42). However, combination of markers requires larger cohorts. We did not assess theses combinations in the present study due to the high risk of overfitting results with too few patients. We also recognize that the relatively small sample size of our study limits our ability to perform further analyses, such as age or gender-stratified analysis, which would require larger cohorts.

In pediatric mTBI, collaborative efforts across multiple centers for the recruitment of larger cohorts are needed to propel our comprehension forward and to consolidate our results. Despite the relatively small sample size, our study is nevertheless representative and generalizable to the entire mTBI pediatric population. Indeed, the presentation of symptoms, the rate of CT scanned patients, the rate of patients with ICI, and the performance obtained for the S100b known blood-based brain injury marker in pediatric were in accordance with the literature (36, 43, 44).

## Conclusion

This study shows that immune dysfunction with increased level of the IL6 inflammatory cytokine is observed in children with mTBI during the acute phase. The diagnostic value of this cytokine must be taken into consideration, as the measurement of its blood level after mTBI can significantly help in the management of patients at the ED. In the studied population, IL6 demonstrated a 100% NPV and was able to identify up to 50% of patients without intracranial injury (ICI) over a longer time window (24 h), compared to more commonly recognized biomarkers such as GFAP, HFABP, and S100b. Its blood measurement can avoid unnecessary CT scan and can contribute to reduce the length of stay in the ED, for children and their families. These results will require further validation in a larger multicenter cohort before clinical application, as well as investigation of a panel of biomarkers, including both brain injury and inflammatory mediators.

## Data availability statement

The raw data supporting the conclusions of this article will be made available by the authors, without undue reservation.

## Ethics statement

The studies involving humans were approved by Commission Cantonale d’Ethique de la Recherche sur l’être humain (CCER). The studies were conducted in accordance with the local legislation and institutional requirements. Written informed consent for participation in this study was provided by the participants’ legal guardians/next of kin.

## Author contributions

A-CC: Conceptualization, Data curation, Formal analysis, Funding acquisition, Investigation, Methodology, Project administration, Resources, Software, Validation, Visualization, Writing – original draft, Writing – review & editing. VP: Conceptualization, Data curation, Investigation, Writing – original draft, Writing – review & editing, Resources. CH: Data curation, Writing – review & editing, Resources. MS: Resources, Writing – review & editing. FR: Resources, Writing – review & editing. FS: Resources, Writing – review & editing. CR: Resources, Writing – review & editing. CK: Conceptualization, Funding acquisition, Investigation, Writing – review & editing. FM: Data curation, Resources, Writing – review & editing. VW: Data curation, Resources, Writing – review & editing. LG: Formal analysis, Writing – review & editing. JM: Writing – review & editing. SM: Conceptualization, Data curation, Funding acquisition, Investigation, Methodology, Project administration, Resources, Supervision, Validation, Visualization, Writing – review & editing. J-CS: Conceptualization, Data curation, Funding acquisition, Investigation, Methodology, Project administration, Resources, Supervision, Validation, Visualization, Writing – review & editing.

## Footnotes

^1^http://www.rporject.org, version 4.3.0.

^2^http://www.rstudio.com, version 2023.06.0.
