# Supplementary material for: Diagnostic potential of IL6 and other blood-based inflammatory biomarkers in mild traumatic brain injury among children
Source: Front Neurol. 2024 Jul 11;15:1432217. doi: 10.3389/fneur.2024.1432217 (PMC11270961; doi:10.3389/fneur.2024.1432217)
Supplement: Supplementary file 1 [file Data_Sheet_1.docx]

**SUPPLEMENTAL DATA**

**Diagnostic potential of IL6 and other of blood-based inflammatory biomarkers in mild traumatic brain injury among children.**

Anne-Cécile Chiollaz^1*^, Virginie Pouillard^2^, Céline Habre^3^, Michelle Seiler^4^, Fabrizio Romano^5^, Fabian Spigariol^6^, Céline Ritter Schenk^7^, Christian Korff^2^, Fabienne Maréchal^8^, Verena Wyss^4^, Lyssia Gruaz^1^, t-BIOMAP investigators and participants, Joan Montaner^9^, Sergio Manzano^10°^, Jean-Charles Sanchez^1°*^.

Summary

[TABLES: 2](#_Toc170823110)

[Table 1. Suppl: Biomarkers expression in CT-scanned and in-hospital-observation mTBI patients (within 24 hours). 2](#_Toc170823111)

[Table 2. Suppl: Biomarkers expression in CT-scanned mTBI patients (within 24 hours). 2](#_Toc170823112)

[Table 3. Suppl: Cytokines best performances to rule-out mTBI patients (within 6 hours). 3](#_Toc170823113)

[Table 4. Suppl: Clinical parameters and IL6 expression in ISOLATED mTBI patients (with or without CT scan) – within 24 hours. 4](#_Toc170823114)

[Table 5. Suppl: IL6 best performances to rule-out isolated mTBI patients (within 24 hours). 4](#_Toc170823115)

[Table 6. Suppl: multivariate regression models with or without ECI (within 24 hours). 5](#_Toc170823116)

[FIGURES: 5](#_Toc170823117)

[Figure 1. Suppl: Biomarkers and age correlation in controls patient (n=74). 5](#_Toc170823118)

TABLES:

## Table 1. Suppl: Biomarkers expression in CT-scanned and in-hospital-observation mTBI patients (within 24 hours).

|  | **CT- or Observation (n=271)** | **CT+ (n=14)** | **P-value** |
| --- | --- | --- | --- |
| **IL6 (pg/ml)** |  |  |  |
| Mean (SD) | 2.37 (7.24) | 5.89 (8.70) | <0.001 |
| Median [Min, Max] | 0.920 [0.0771, 77.6] | 2.62 [0.856, 32.3] |  |
| Missing | 2 (0.7%) | 0 (0%) |  |
| **IL8 (pg/ml)** |  |  |  |
| Mean (SD) | 44.6 (354) | 183 (636) | 0.339 |
| Median [Min, Max] | 9.96 [1.84, 5640] | 12.6 [3.96, 2390] |  |
| Missing | 2 (0.7%) | 0 (0%) |  |
| **IL10 (pg/ml)** |  |  |  |
| Mean (SD) | 1.47 (6.10) | 1.56 (2.37) | 0.062 |
| Median [Min, Max] | 0.430 [0.0634, 92.2] | 0.640 [0.205, 9.37] |  |
| Missing | 1 (0.4%) | 0 (0%) |  |

*p* values correspond to the Mann-Whitney U test comparison.

## Table 2. Suppl: Biomarkers expression in CT-scanned mTBI patients (within 24 hours).

|  | **CT- (n=48)** | **CT+ (n=14)** | **P-value** |
| --- | --- | --- | --- |
| **IL6 (pg/ml)** |  |  |  |
| Mean (SD) | 5.04 (11.9) | 5.89 (8.70) | 0.165 |
| Median [Min, Max] | 1.79 [0.115, 62.7] | 2.62 [0.856, 32.3] |  |
| **IL8 (pg/ml)** |  |  |  |
| Mean (SD) | 166 (827) | 183 (636) | 0.337 |
| Median [Min, Max] | 15.6 [4.35, 5640] | 12.6 [3.96, 2390] |  |
| **IL10 (pg/ml)** |  |  |  |
| Mean (SD) | 3.64 (13.6) | 1.56 (2.37) | 0.874 |
| Median [Min, Max] | 0.932 [0.0634, 92.2] | 0.640 [0.205, 9.37] |  |

*p* values correspond to the Mann-Whitney U test comparison.

## Table 3. Suppl: Cytokines best performances to rule-out mTBI patients (within 6 hours).

| **Variable** | **Sensibility (%)**  **(95%CI)** | **Specificity (%)**  **(95%CI)** | **Threshold (pg/ml)** | **AUC**  **(95%CI)** |
| --- | --- | --- | --- | --- |
| IL6 | 100  (100 – 100) | 50.00  (43.27 – 56.73) | 0.85 | 76.5  (63.1 – 89.8) |
| IL10 | 100  (100 – 100) | 10.24  (6.16 – 14.32) | 0.20 | 68.5  (42.1 – 95.0) |
| IL8 | 100  (100 – 100) | 4.41  (1.65 – 7.17) | 3.91 | 60.00  (32.1 – 87.8) |

**(A)**

**(B)**

| **Variable** | **Sensibility (%)**  **(95%CI)** | **Specificity (%) (95%CI)** | **Threshold (pg/ml)** | **AUC**  **(95%CI)** |
| --- | --- | --- | --- | --- |
| IL6 | 100  (100 – 100) | 18.18  (6.08 – 30.28) | 0.82 | 54.00  (31,1 – 77.0) |
| IL10 | 100  (100 – 100) | 3.03  (-2.35 – 8.41) | 0.20 | 48.00  (18.0 – 77.9) |
| IL8 | 100  (100 – 100) | 0.00  (0,00 – 0,00) | -Inf | 40.40  (11.6 – 69.2) |

1. **Best performances to rule out a maximum CT– and in-hospital-observation patients, while all CT+ patients have been identified** (CT-scanned and observed without CT [>6hours at ED] patients). IL6 yields the best performances (100% SE - 50% SP)
2. **Best performances to rule out a maximum of CT– patients, while all CT+ patients have been identified** (only CT-scanned patients). IL6 yields the best performances (100% SE - 18% SP)

AUC=area under the curve

## Table 4. Suppl: Clinical parameters and IL6 expression in ISOLATED mTBI patients (with or without CT scan) – within 24 hours.

|  | **Isolated mild TBI n=244** | | |  |
| --- | --- | --- | --- | --- |
|  | **in-hospital obs. n=199** *(82%)* | **CT**  **n=45** *(18%)* | |  |
|  |  | **CT- n=35** *(78%)* | **CT+ n=10** *(22%)*  *(4% of mTBI)* | **P-value** |
| **Age (yo)** |  |  |  |  |
| Mean (SD) | 8.46 (4.39) | 8.12 (4.63) | 7.56 (4.69) | 0.809 |
| Median [Min, Max] | 9.02 [0.20, 15.9] | 7.50 [0.10, 15.8] | 7.15 [0.90, 13.5] |  |
| **Sex, n (%)** |  |  |  |  |
| Boys | 111 (55.8%) | 21 (60.0%) | 8 (80.0%) | 0.301 |
| **Severity of injury, n (%)** |  |  |  |  |
| GCS 14 | 14 (7.0%) | 10 (28.6%) | 2 (20.0%) | <0.001 |
| GCS 15 | 185 (93.0%) | 25 (71.4%) | 8 (80.0%) |  |
| **Skull fracture (on CT), n (%)** |  |  |  |  |
| Simple skull fracture (not PECARN criterion) | - | 6 (17.1%) | 8 (80.0%) | <0.001 |
| **Time laps TBI-blood (hours)** |  |  |  |  |
| Mean (SD) | 6.16 (4.34) | 6.66 (5.53) | 9.10 (7.80) | 0.52 |
| Median [Min, Max] | 5.00 [1.00, 23.0] | 5.00 [1.00, 24.0] | 8.00 [2.00, 24.0] |  |
| **IL6 (pg/ml)** |  |  |  |  |
| Mean (SD) | 1.39 (2.42) | 5.48 (13.8) | 7.05 (10.0) | <0.001 |
| Median [Min, Max] | 0.712 [0.07, 23.2] | 1.66 [0.11, 62.7] | 2.77 [1.32, 32.3] |  |
| *Missing* | *2 (1%)* | *0 (0%)* | *0 (0%)* |  |

*p* values correspond to the Kruskall-Wallis test comparison (non-parametric ANOVA).

## Table 5. Suppl: IL6 best performances to rule-out isolated mTBI patients (within 24 hours).

| **Variable** | **Groups (n)** | | **Sensibility (%)**  **(95%CI)** | **Specificity (%) (95%CI)** | **Threshold (pg/ml)** | **AUC**  **(95%CI)** |
| --- | --- | --- | --- | --- | --- | --- |
| IL6 | **CT-/Obs**  234 | CT+  10 | 100  (100 – 100) | 68  (62.15 – 73.85) | 1.32 | 70.9  (54.9-86.8) |
|  | **CT-**  35 | CT+  10 | 100  (100 – 100) | 49  (34.39– 63.61) | 1.26 | 84.8  (76.5-93.0) |

## Table 6. Suppl: Multivariate regression models with or without ECI (within 24 hours).

| **Variables** | **Groups (n)** | | | **Unadjusted OR (CI 95%)** | | **Adjusted OR**  **(CI 95%)** | **P value** |
| --- | --- | --- | --- | --- | --- | --- | --- |
|  | **CT-/Obs**  **271** | **CT+**  **14** | |  |  |  |  |
| IL6 |  | |  | | 1.01 (0.94 - 1.06) | 1.05 (0.99 - 1.10) | 0.046 |
| ECI |  |  |  |  | 1.08 (0.26 - 3.88) | 3.32 (0.79 - 14.02) | 0.079 |

# FIGURES:

##
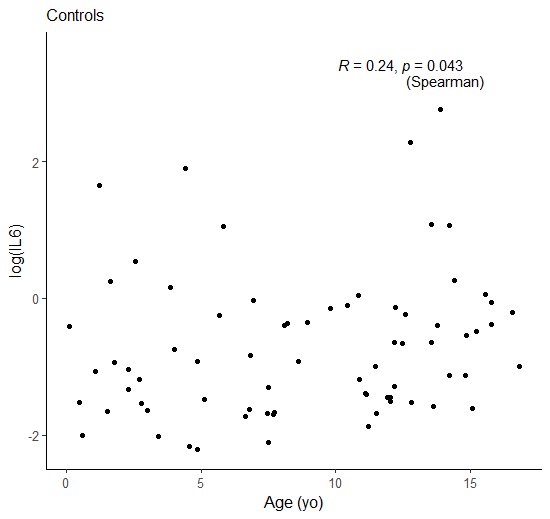

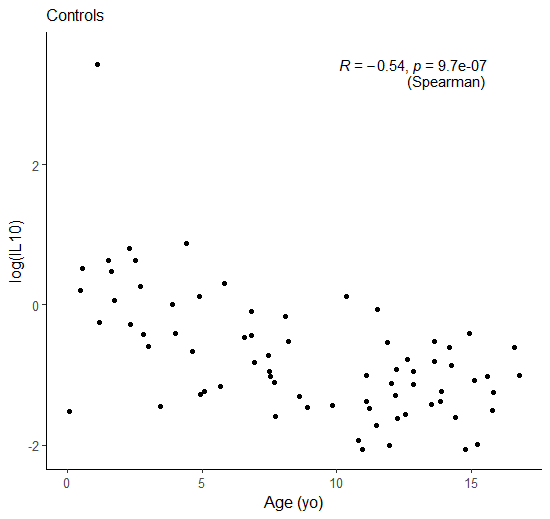

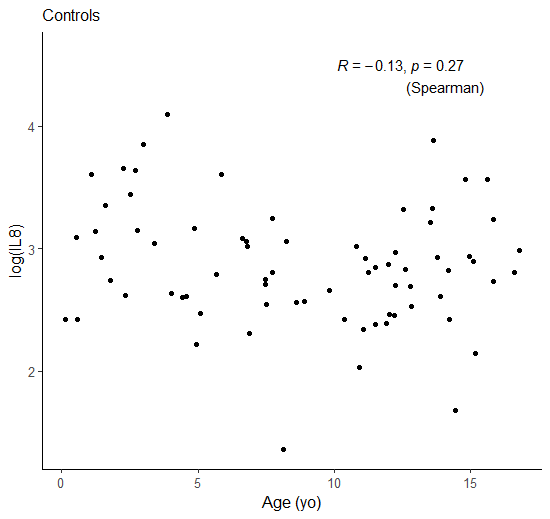
Figur**e 1. Suppl: Biomarkers and age correlation in controls patient (n=74).**
